# Parliament and the Profession

**Published:** 1916-07-29

**Authors:** 


### PARLIAMENT AND THE PROFESSION.

### Artificial Limbs from America.

Have orders for the supply of artificial limbs to wounded soldiers been given to American firms? This in effect was a question which Mr. W. Thorne put to the Financial Secretary to the War Office. Mr. Forster's reply was that orders had been placed with sixteen different firms, of which three are American and the remainder British. The orders, he added, are placed in accordance with the recommendations of the consulting surgeons at Roehampton, and the limbs supplied are of the types commended by the judges at the exhibition held there in July a year ago. The prices range from £6 10s. to £17 for an artificial arm, and from £8 18s. 6d. to £20 for an artificial leg, the amounts varying according to the nature of the amputation.

### Private Practitioners and Medical Boards.

In a question he put to the representative of the War Office, Mr. Edmund Harvey suggested that certain Army Medical Boards passed over evidence submitted by civilian practitioners and passed for general service, after brief examination, men who subsequently broke down under training. Mr. Forster replied that boards had been instructed to give due consideration to medical evidence submitted by civilian practitioners, and it was not the case that there had been any general failure to do this; in view, however, of statements which have been made, attention has again been called to the point.

### Massage in Military Hospitals.

Mr. Forster, replying to a question by Mr. Tyson Wilson, stated that arrangements had been made for an official inspection of the schools connected with the National Association of Trained Masseuses and Masseurs. Until the results of the inspection are available, it will not be possible to say whether the same recognition will be given to this Association as is given to the Almeric Paget Massage Corps.

### Nerve-Shaken Officers.

A question which Mr. Forster thought was based upon a misapprehension was asked by Mr. King. The question inferred that the Prince of Wales's National Relief Fund had voted £10,000 to Lord Knutsford's hospitals for nerve-shaken officers, "so that such cases may be treated apart from lunacy asylums and lunacy administration." Mr. King asked whether the Government would provide the necessary funds so that nerve-shaken sailors and soldiers might be similarly treated apart from asylums. The reply was that soldiers suffering from nerve-shock are treated in military hospitals under the control of the War Office, and that the arrangements made for these cases had been pronounced highly satisfactory by various independent and competent judges.

### Hospital Accommodation for Sailors.

In a series of questions asked by Sir A. Williamson it was suggested that there might not perhaps be complete harmony between the Admiralty and the War Office with regard to hospital accommodation for wounded sailors and soldiers. Thus he asked if there was any arrangement by which wounded sailors could be sent to hospitals where beds which were reserved for the Army were empty. Dr. Macnamara assured the hon. member that complete harmony existed between the two departments.

### After-treatment of Wounded Soldiers.

Replying to Mr. Hume-Williams, Mr. Forster stated that soldiers discharged from the Army on account of wounds could, on application to the Secretary of the Royal Hospital, Chelsea, obtain treatment in a military hospital, provided there was a reasonable probability that their disability would be cured or materially improved thereby.

### Promotions in the R.A.M.C.

One hundred and fifty-five captains in the R.A.M.C. have been promoted since the beginning of the war. In making a statement to this effect Mr. Forster added that the number of promotions in the Royal Army Medical Corps had not been fixed so as to bear a definite proportion to combatant promotions of similar rank.

### Sale of Cocaine to Soldiers.

The sale of cocaine to any member of the Forces is already prohibited by an Order under the Defence of the Realm Regulations, and the Home Secretary announced that it was intended to obtain at once an extension of the Regulation to enable the Government to control the sale of the drug generally.

### Treatment of Acute Mental Cases.

Replying to Commander Bellairs, Dr. Macnamara explained that while cases of neurasthenia in the Royal Navy were treated in general medical wards, with excellent results up to the present, acute mental cases were treated in the special mental wards provided in our three large naval hospitals. The increase in the percentage of acute mental cases in the Navy since the war began has not been very great.

